# Evaluation of the accuracy of a surface-guided radiotherapy system for patient positioning in radiotherapy of breast cancer

**DOI:** 10.1016/j.phro.2026.100933

**Published:** 2026-02-19

**Authors:** Gracinda Johansson, Johan Knutsson, Martin Olin

**Affiliations:** a Medical Physics, Diagnostics and Technology, Södersjukhuset, Stockholm, Sweden; b Department of Clinical Science and Education, Karolinska Institutet, Stockholm, Sweden

**Keywords:** Breast cancer radiotherapy, Surface-guided radiotherapy, Patient positioning, Position verification

## Abstract

**Background and Purpose:**

The accuracy of a surface-guided radiotherapy (SGRT) system for patient positioning in free-breathing breast cancer radiotherapy (RT) was evaluated against cone-beam computed tomography (CBCT)-based image registration.

**Materials and Methods:**

A total of 106 treatment fractions from 20 patients who received breast RT in free-breathing in our institution were analysed. Patient positioning was performed using an SGRT system, without initial alignment to skin markers. The positioning deviations were reduced to within our clinical tolerance of ± 3.0 mm (translations) and ± 3.0° (rotations) before CBCT acquisition. CBCT-based setup corrections were calculated using bony anatomy registration between the CBCT and planning computed tomography (CT) images. The translational and rotational corrections derived from SGRT were compared with those from CBCT. A two-sample paired *t*-test with a significance level of 0.05 was performed.

**Results:**

No significant differences were observed between SGRT and CBCT for longitudinal (*p* = 0.42) and lateral translations (*p* = 0.70). Vertical translation and rotations about all three axes were within clinical tolerance levels with SGRT, while CBCT-based corrections showed statistically significant differences (*p* < 0.05).

**Conclusions:**

The results support the feasibility of marker-free SGRT with CBCT verification as a reliable setup method in free-breathing breast cancer RT.

## Highlights

• Accuracy of surface-guided positioning evaluated for breast cancer radiotherapy.
• Deviations within 3 mm/3° in all directions after surface matching.
• Surface scanning found to be a reliable setup method for breast cancer radiotherapy.

## Graphical abstract

**Figure:**
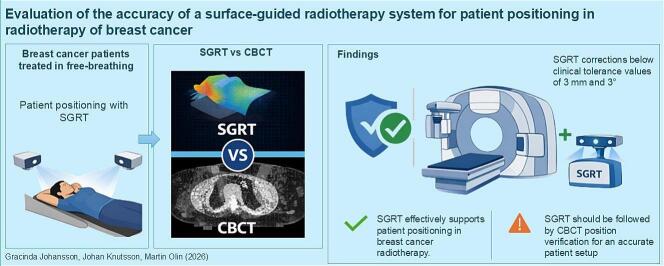

## 1 Introduction

Breast cancer radiotherapy (RT) is typically delivered through external beam irradiation [1]. To be able to deliver high tumoricidal doses to target volumes while minimizing the dose given to organs at risk (OAR), accurate patient positioning is required. There is also a need that the patient position is consistent and reproducible throughout the delivery of all treatment fractions [1], [2]. Patient setup in RT has traditionally been performed by initially aligning tattoos/markers placed on the patient skin to the room lasers, followed by the acquisition of two- or three-dimensional kilovolt (kV)/megavolt (MV) X-ray images for the setup verification [3], [4]. Recent technological advances and their integration into RT have facilitated the clinical implementation of surface-guided radiation therapy (SGRT) [5], [6]. The use of SGRT provides a non-invasive, fast and accurate means of patient positioning in RT of various tumour sites. It can also be used for real-time motion monitoring during the delivery of a treatment fraction and in gated RT [5] e.g., deep inspiration breath hold (DIBH) in breast cancer RT. The evaluation of the use of the SGRT for patient positioning in breast cancer RT in comparison with laser alignment to skin markers has been performed [5], [7], [8], [9], [10]. The use of SGRT in most of these studies is based on an initial alignment of skin markers to the room lasers, with a limited number of studies based on an independent use of SGRT in patient positioning, e.g., the study by Jimenez et al. [11], [12], [13], [14]. The aim of this study is to evaluate the accuracy of an SGRT system when used alone for patient positioning in RT of breast cancer, compared to image registration between cone-beam computer tomography (CBCT) and computer tomography (CT).

## 2 Material and methods

A total of 106 RT sessions of 20 breast cancer patients (4 left-sided and 16 right-sided breast cancer) that received treatment in three consecutive weeks in May of 2021 at Stockholm South Hospital (Stockholm, Sweden), were evaluated. These patients received adjuvant whole breast RT following breast conserving surgery. The delineation of structures is presented in the Supplementary Material. The prescription dose was 2.67 Gy in 15 fractions to a total dose of 40.05 Gy. Treatment planning was based on a three-dimensional conformal therapy (3D-CRT) technique using tangential fields. For every treatment fraction, these patients were positioned in free breathing (FB) followed by daily CBCT acquired also in FB. The daily surface scan used for patient positioning was matched to the reference body surface, which was imported from the treatment planning system (TPS). The reference image was cropped to only include the volume of interest relevant for the treatment, extending longitudinally from the patient’s neck down to include the two breasts and approximately 5 cm below the caudal end of the breasts. Patient positioning was performed with the help of the Catalyst system (C-RAD AB, Uppsala, Sweden) consisting of three cameras, one frontal and two oblique-lateral. The patient was initially positioned lying on the treatment couch in the treatment position. The C-4D software (C-RAD AB, Uppsala, Sweden) displays both the patient reference and live surfaces. The correct patient position was achieved when these two images matched the best. The patient couch, which allows for three degrees of freedom (3DoF) corrections only, was manually moved to a position which resulted in the remaining relative translation corrections within 5 cm. Thereafter, automatic couch movements were used to minimise the relative translations shown in the C-4D software to within the tolerance levels for the three translational axes (±3 mm). The C-4D software also shows the rotational deviations of the live surface in relation to the reference. These errors were also corrected for, with a tolerance level of ± 3°. The correction for posture errors was then performed by adjusting the patient aiming to remove the colour projections that indicate high or low regions in the live surface in relation to the reference surface. A threshold value of 7 mm was used to show the deviations. The resulting body surface scan was thereafter taken as the reference for the actual treatment fraction and used to monitor patient motion during the current fraction.

The CBCT image acquisition was performed using CBCT presets with a gantry rotation of 190° (counterclockwise from 40° to 210° for right breast and clockwise from 320° to 150° for left breast). The volume of interest (i.e., the clipbox) used for CBCT-CT image registration was defined to include as much planning target volume (PTV) volume as possible. Medially, the clipbox should include a large part of the sternum; cranially and dorsally, the clipbox was defined to include the vertebral column. The center of the clinical target volume (CTV) was chosen as the correction reference point. The possible corrections for translations and rotations from the CBCT were obtained in 6DoF, with the help of the XVI Synergy system (Elekta AB, Stockholm, *Sweden*). Image registration preset with bone registration, accounting for translations and rotations, was used. A clinical tolerance of ± 3° was used for rotations in the CBCT-CT image registration. Combined translational and rotational corrections were reduced to translational components only, due to the treatment couch being limited to movements in 3DoF. A visual inspection of the resulting image registration was performed, before the registration was accepted for use in the actual treatment fraction.

The CBCT–CT image registration was used as the reference standard for pre-treatment position verification. Patient positioning accuracy was evaluated solely for the SGRT system. To assess concordance between SGRT and the reference standard, the final translational and rotational offset corrections generated by the SGRT system prior to CBCT acquisition were extracted and compared with the corresponding CBCT–CT registration results. A two-sample paired *t*-test, with a significance level of 0.05 was carried out. A Bland-Altman analysis was also used to assess the agreement between SGRT- and CBCT-derived positioning corrections.

## 3 Results

**Fig. 1:**
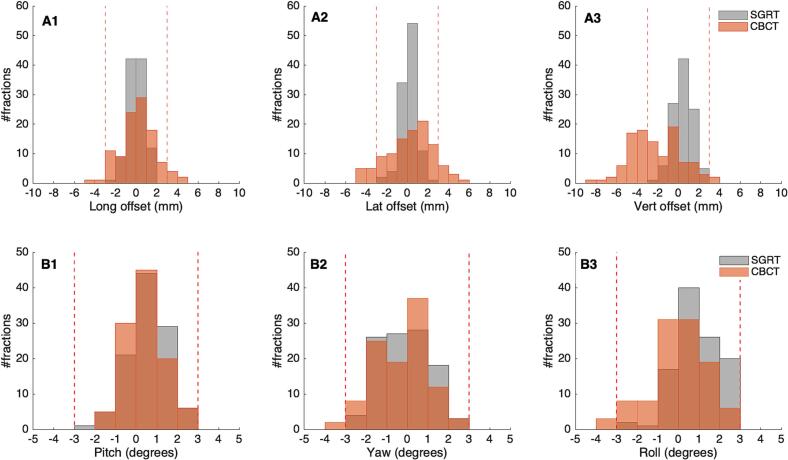
Distribution of the translational (A1-3) and rotational (B1-3) corrections obtained with the SGRT system (grey) and CBCT-CT image registration (orange). The vertical red lines represent the clinical tolerance levels of ± 3 mm for translations and ± 3° for the rotational offsets. (For interpretation of the references to colour in this figure legend, the reader is referred to the web version of this article.)

All translational and rotational values recorded with the SGRT system following patient positioning were within our predefined clinical tolerance limits. These values were subsequently compared to the corrections derived from the CBCT. No statistically significant differences were observed between the mean translational corrections in the longitudinal (*p* = 0.42) and lateral (*p* = 0.70) directions. In contrast, a significant difference was detected in the vertical direction (*p* < 0.001) between the SGRT system and CBCT-CT image registration. For rotational corrections, statistically significant differences were found for pitch (*p* = 0.03), yaw (*p* = 0.04), and roll (*p* < 0.001). A detailed summary of the results is presented in Table 1, while Fig. 1 provides a graphical representation of both translational and rotational corrections. The results of the Bland-Altman analysis are presented in the Supplementary Material.

**Table 1:** Mean and standard deviation for the rotational and translational corrections obtained with the SGRT and XVI systems.

		SGRT	CBCT	*p-*value
Translational corrections (mm)	Longitudinal	−0.03 (±0.84)	0.13 (±1.64)	0.42
	Lateral	0.12 (±0.79)	0.22 (±2.22)	0.70
	Vertical	0.45 (±0.95)	−2.20 (±2.45)	0
Rotational corrections (degrees)	Pitch	0.54 (±0.93)	0.39 (±0.91)	0.03
	Yaw	−0.19 (±1.12)	−0.37 (±1.26)	0.04
	Roll	0.83 (±1.12)	−0.06 (±1.36)	0

## 4 Discussion

In this study, the accuracy of an SGRT system for patient positioning in tangential breast cancer RT was evaluated. Translational and rotational setup deviations recorded by the SGRT system were compared with those obtained from CBCT-based image registration. Our results demonstrated a good overall agreement between the two methods for longitudinal and lateral translations, with mean differences near zero and non-significant *p*-values (Table 1). However, statistically significant differences were observed in the vertical direction and for rotations around all directions.

In the vertical direction, all SGRT-derived corrections were within the predefined clinical tolerance of 3 mm for patient setup. However, 44% of fractions required corrections by more than 3 mm with CBCT. This pattern suggests the presence of a systematic discrepancy between SGRT positioning and CBCT verification in the vertical direction. These findings are consistent with previously published studies that evaluated the use of SGRT for breast cancer patient positioning [15], [16]. One plausible explanation is a change in patient posture between the planning CT and treatment delivery, with patients being more relaxed during treatment [15]. Such relaxation may lead to a consistent shift that is subsequently corrected by CBCT toward more negative vertical values relative to the initial SGRT positioning. Mankinen et al. [16] suggested that the bias in the vertical direction could be due to respiratory phase differences and intra-fraction motion. Additionally, Hattel et al. [15], found that setups using room lasers were more accurate in the vertical direction compared to SGRT systems. Regarding rotational corrections across all three axes, the values suggested by the SGRT system remained within ± 3° across all treatment fractions. This resulted in CBCT corrections below ± 3° in 100%, 98.1%, and 97.2% of the studied treatment fractions for pitch, yaw, and roll, respectively. In instances where CBCT rotations exceeded tolerance, the resulting image registration was evaluated to decide whether to accept the registration and proceed with treatment or reposition the patient and acquire a new CBCT image.

In our institution, the patient positioning is performed using solely the SGRT system, followed by a CBCT image verification. Differently from other studies in this area [8], [9], [10], the conventional patient positioning using room lasers was not performed. Crop et al. [8] compared between the use of room lasers, an SGRT system and a mega-voltage CT (MVCT) imaging for patient positioning in postoperative RT of breast cancer. This study only reported the rotational correction for roll, apart from the three-dimensional (3D) translational corrections between the three positioning modalities evaluated. It was found that the use of SGRT resulted in a smaller setup error compared to when room lasers were used, for both the translational and rotational error corrections. The corrections for the roll deviations were 1.1 and 0.9° (68% confidence interval), respectively with the laser-based positioning and the SGRT system, compared to the MVCT. In respect to the roll deviations observed in our study, 64.5% of all treatment fractions exhibited roll deviations of 1° or less after CBCT correction of SGRT-based patient positioning.

According to the results obtained in this study, most of the CBCT-suggested rotational corrections were well below our tolerance levels for the rotational deviations. It also resulted in few cases where repositioning was required. This clearly indicates that the surface scanning system, without initial positioning with room lasers, could be used to achieve a high level of positioning accuracy for breast cancer patients, as already suggested in the studies by, e.g., Wei et al. [10] and Kügele et al. [7]. Importantly, this does not replace the need for daily radiographic imaging to verify patient setup. Wei et al. [10] analysed the effectiveness of the use of a surface scanning system vs. CBCT imaging for setup corrections in breast cancer RT. They found a good correlation between these two modalities, which led the authors to suggest the use of the SGRT system without CBCT verification. A contradiction to this assumption was, however, presented in the study by Hattel et al. [15] and Mankinen et al. [16], where it was found that despite the improvements in patient positioning with the use of a surface scanning system, it should be followed by planar or 3D X-ray imaging. A feasibility study on a tattoo-free setup in breast cancer RT [11] found that a similar level of accuracy with reduced setup times could be obtained with the tattoo-free setup, compared to when skin markers were used, provided that a 3D surface imaging and a verification planar X-ray imaging was performed.

The SGRT system studied, makes use of a deformable registration algorithm [17] to calculate the isocenter position and the required corrections to reach the best possible match between the daily live surface and the reference patient surface. Patient positioning with surface scanning is based on the information obtained from the patient skin and does not necessarily offer information about the internal patient anatomy. For this reason, it is still important that SGRT is followed by some form of position verification (e.g. CBCT). Following the results of the present study, our clinic has adopted the SGRT system for patient positioning in RT across additional treatment sites requiring precise alignment. In conclusion, markerless SGRT effectively supports patient positioning. However, it should be followed by position verification with X-ray imaging for an accurate patient setup and a better treatment position reproducibility throughout the course of RT.

## 5 Declaration of generative AI and AI-assisted technologies in the manuscript preparation process

During the preparation of this work the authors used ChatGPT to create the illustrations used for the preparation of the graphical abstract. After using this tool, the authors reviewed and edited the content as needed and take full responsibility for the content of the published article.

## CRediT authorship contribution statement

**Gracinda Johansson:** Conceptualization, Data curation, Formal analysis, Investigation, Methodology, Writing – original draft, Writing – review & editing, Software, Visualization. **Johan Knutsson:** Data curation, Formal analysis, Methodology, Writing – review & editing. **Martin Olin:** Data curation, Formal analysis, Methodology, Writing – review & editing.

## Declaration of competing interest

The authors declare that they have no known competing financial interests or personal relationships that could have appeared to influence the work reported in this paper.
