# Supplementary material for: Evaluation of the accuracy of a surface-guided radiotherapy system for patient positioning in radiotherapy of breast cancer
Source: Phys Imaging Radiat Oncol. 2026 Feb 19;37:100933. doi: 10.1016/j.phro.2026.100933 (PMC12950478; doi:10.1016/j.phro.2026.100933)
Supplement: Supplementary Data 1 [file mmc1.docx]

**Supplemental materials**

Supplemental material A

**Delineation of structures and treatment planning**

A computed tomography (CT) image, which was used for planning the radiotherapy (RT) treatment, was acquired on a Siemens Somatom Definition Edge (Siemens Healthineers AG, Erlangen, Germany), with the patient lying on the table in head-first, supine position. A Wing Boards^TM^ (CIVCO Radiotherapy, Iowa, USA) was used to immobilise the patient in a reproducible position for all treatment fractions. CT image sets consisting of 3 mm axial slices were used for treatment planning.

The clinical target volume (CTV) was delineated by an oncologist, to include the whole breast, following institutional guidelines. The surgical clips which were implanted following breast conserving surgery were delineated. These were subsequentially used during the CBCT-CT matching prior to treatment delivery. The planning target volume (PTV) was then created by adding a uniform CTV to PTV margin of 7 mm. Both the CTV and the PTV were cropped 5 mm under the body surface to exclude the electron build-up region of the photon depth-dose distribution in the dose statistics of the target volumes. The OARs (ipsilateral lung and the heart) were also delineated.

The treatment plans were prepared in Monaco (Elekta AB, Stockholm, Sweden) treatment planning system (TPS). The conventional modality used for treatment planning is the three-dimensional conformal therapy (3D-CRT). The plan is prepared with tangential fields for breast treatments. For specific cases, where it was not possible to reach a satisfactory PTV dose coverage while keeping the dose given to the OARs in clinical acceptable levels, techniques such as the forward-planned intensity modulated radiation therapy (IMRT) was used to prepare the treatment plans. The treatment was delivered in a Versa HD (Elekta AB, Stockholm, Sweden) linear accelerator.

Supplemental material B

**Bland-Altman analysis**

The agreement between the SGRT- and CBCT-derived translational and rotational corrections was assessed using Bland–Altman analysis. For each degree of freedom, the mean difference (bias) and the 95% limits of agreement were calculated by plotting the difference between the two methods against their mean. This analysis was used to quantify systematic offsets and the extent of random variation between SGRT and CBCT for both translational and rotational setup parameters. The results are given in Figure S1 and S2.

Bland–Altman analysis demonstrated good agreement between SGRT and CBCT for all translational and rotational directions, with mean differences close to zero. The limits of agreement were within predefined clinical tolerance levels for the pitch and yaw rotations. A systematic bias was observed in the vertical translation, consistent with the directional differences identified in the statistical comparison.


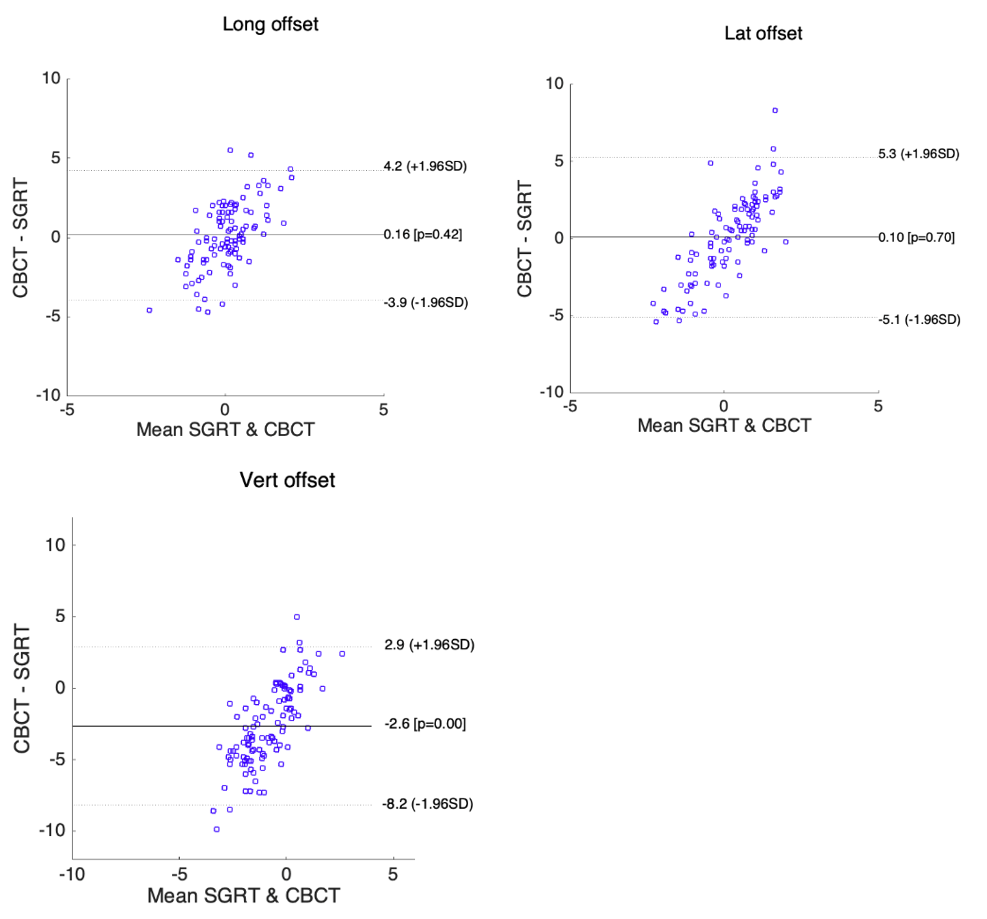


**Figure S1.** Bland-Altman plot for the translational offsets in the longitudinal, lateral and vertical direction.


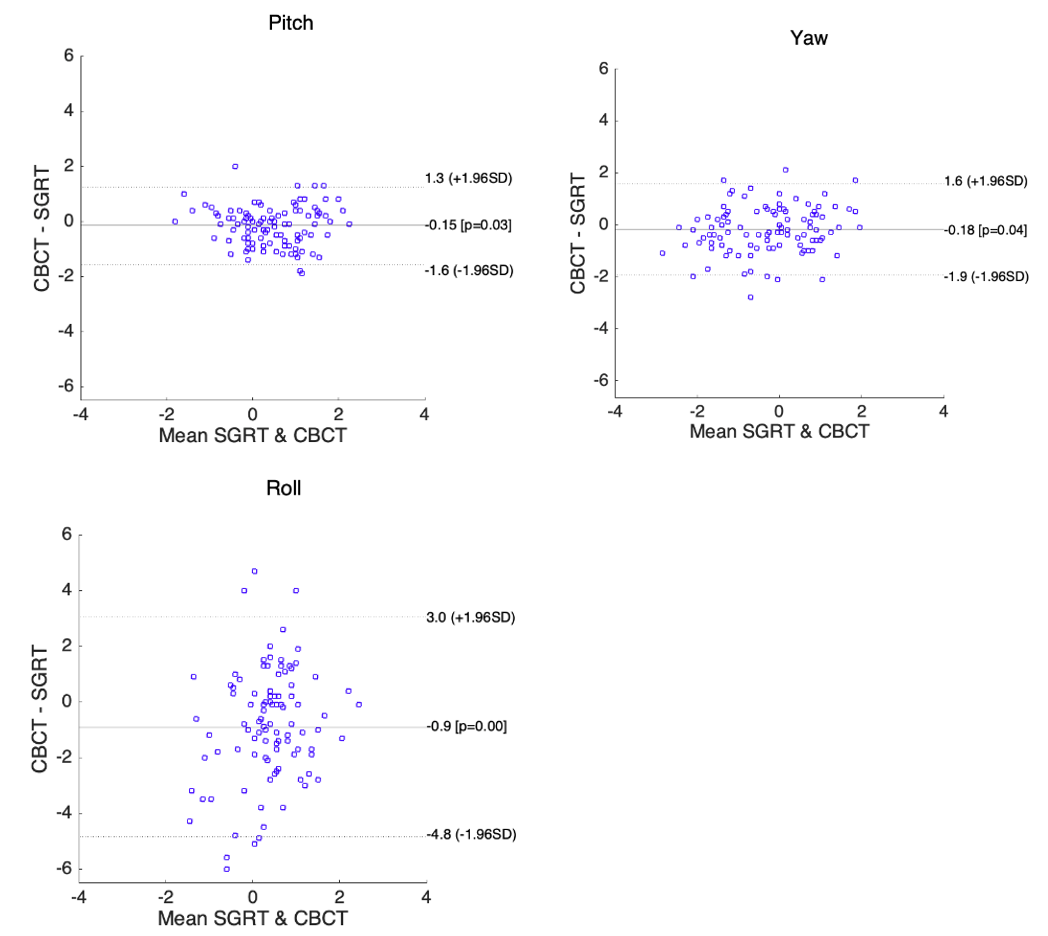


**Figure S2.** Bland-Altman plot for the rotational corrections in three degrees of freedom.
